# Stereochemical investigations on the biosynthesis of achiral (*Z*)-γ-bisabolene in *Cryptosporangium arvum*

**DOI:** 10.3762/bjoc.15.75

**Published:** 2019-03-27

**Authors:** Jan Rinkel, Jeroen S Dickschat

**Affiliations:** 1 Kekulé-Institute for Organic Chemistry and Biochemistry, University of Bonn, Gerhard-Domagk-Str. 1, 53121 Bonn, Germany

**Keywords:** biosynthesis, carbocation chemistry, enzyme mechanisms, nerolidyl diphosphate, terpenes

## Abstract

A newly identified bacterial (*Z*)-γ-bisabolene synthase was used for investigating the cyclisation mechanism of the sesquiterpene. Since the stereoinformation of both chiral putative intermediates, nerolidyl diphosphate (NPP) and the bisabolyl cation, is lost during formation of the achiral product, the intriguing question of their absolute configurations was addressed by incubating both enantiomers of NPP with the recombinant enzyme, which resolved in an exclusive cyclisation of (*R*)-NPP, while (*S*)-NPP that is non-natural to the (*Z*)-γ-bisabolene synthase was specifically converted into (*E*)-β-farnesene. A hypothetical enzyme mechanistic model that explains these observations is presented.

## Introduction

**Figure 1:**
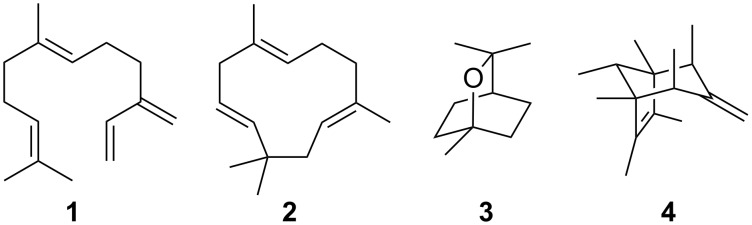
Structures of achiral terpenes: (*E*)-β-farnesene (**1**), α-humulene (**2**), 1,8-cineol (**3**) and sodorifen (**4**).

Given the enormous impact of chirality within biomolecules for all forms of life, it is fascinating to see how nature is able to maintain and reproduce stereochemical information. This concept largely involves the introduction of stereocentres to achiral starting materials by the action of enzymes. While reactions fulfilling this category are still challenging within synthetic chemistry, and methods managing to reach this goal are desperately desired, in the enzymatic world with its completely chiral environment these transformations are ubiquitous, which diminishes the hard border between achiral and chiral. One intriguing example for this kind of reactivity is represented by terpene synthases (TSs), arguably building up the class of natural products with the highest density of stereochemical information, the terpenes. By providing a defined cavity including its molecular coating together with binding and activation of the diphosphate (OPP) moiety, these enzymes convert simple achiral oligoprenyl diphosphates into often complex, polycyclic hydrocarbons or alcohols with introduction of multiple stereocentres in just one enzymatic step [1–3]. With this approach, nature makes perfect use of the versatile chemistry of carbocations with its hydride or proton shifts and Wagner–Meerwein rearrangements leading to a large variety of possible structures. Among terpenoid natural products, achiral compounds are rarely found, but still present. In this group, there are acyclic compounds like the linear sesquiterpene (*E*)-β-farnesene (**1**, Figure 1), which is known as an alarm pheromone in aphids [4–5], but also monocyclic terpenes like α-humulene (**2**), a widely occurring sesquiterpene in many essential oils [6–7]. Whereas the stereochemical imprint of a TS on achiral products is not directly visible, there still can be a chiral cyclisation cascade behind these terpenes. This is true as well for examples featuring a mirror plane like the monoterpene 1,8-cineol (eucalyptol, **3**), for which the absolute configuration of the intermediary terpinyl cation has been investigated using deuterium labelling, demonstrating different stereochemical courses in the plant *Salvia officinalis* [8–9] and in the bacterium *Streptomyces clavuligerus* [10]. Also the highly unusual methylated sesquiterpene sodorifen (**4**) possesses a mirror plane [11] making any labelling experiment hard to interpret and is nevertheless most likely biosynthesised through chiral intermediates [12]. For these cases, it is a particular challenge to uncover the stereochemical information hidden behind the achiral product structure. In this study, we addressed the chiral intermediates in the biosynthesis of the achiral sesquiterpene (*Z*)-γ-bisabolene (**5**) by a TS from a soil bacterium.

## Results and Discussion

### Functional characterisation of a bacterial (*Z*)-γ-bisabolene synthase

**Figure 2:**
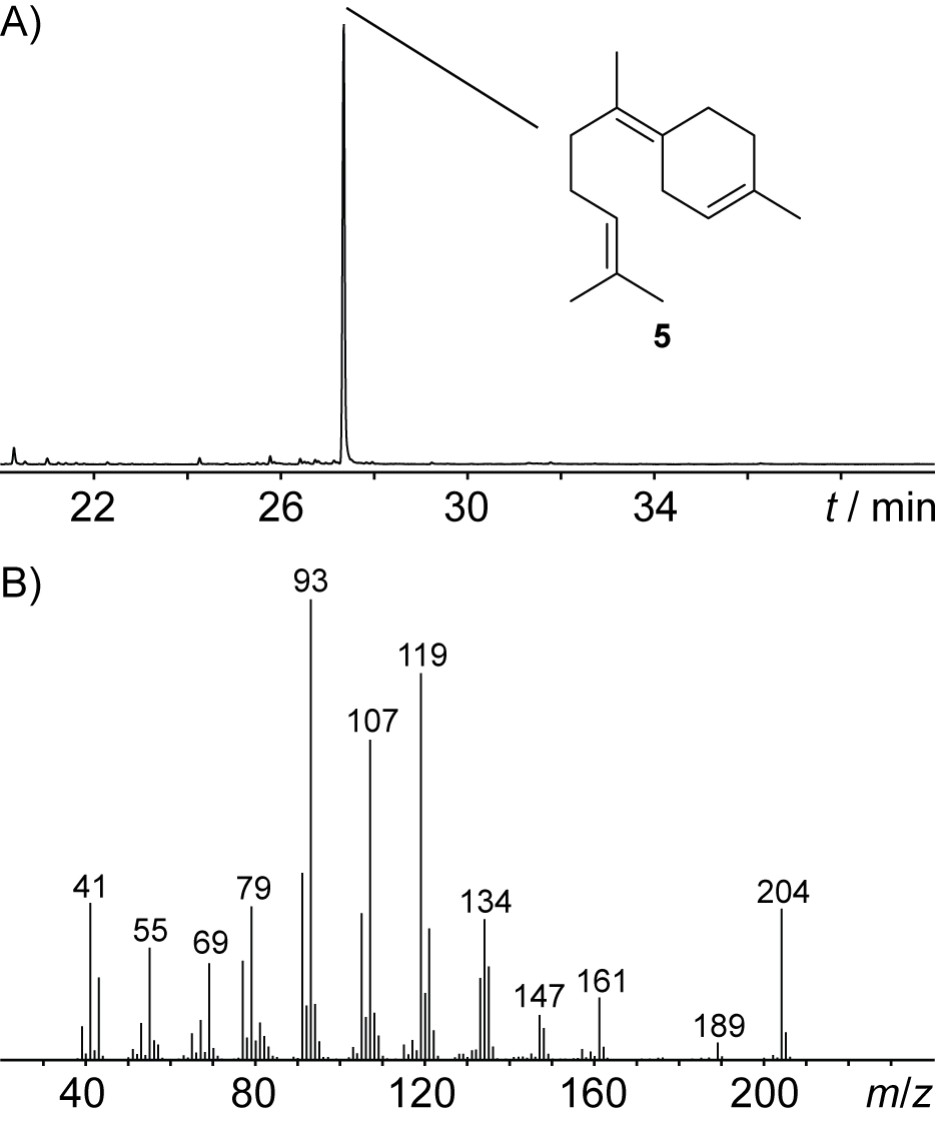
A) Total ion chromatogram of a hexane extract from the incubation of FPP with BbS and B) EI mass spectrum of the main product identified as (*Z*)-γ-bisabolene (**5**).

Within our efforts to characterise bacterial TSs with new functions and mechanisms, a TS (WP_035857999) from the soil actinomycete *Cryptosporangium arvum* DSM 44712 was cloned into the *E. coli* expression vector pYE-express [13] (Table S1, Supporting Information File 1), because of its phylogenetic distance to characterised TSs (Figure S1, Supporting Information File 1). The amino acid sequence of the enzyme features known conserved motifs both for binding [14] and activation [15] of the diphosphate moiety together with structurally important residues [16–17] (Figure S2, Supporting Information File 1). For in vitro activity testing, the enzyme was expressed in *E. coli* BL21(DE3), purified (Figure S3, Supporting Information File 1) and incubated with the common terpene precursors geranyl- (GPP, C_10_), farnesyl- (FPP, C_15_), geranylgeranyl- (GGPP, C_20_) and geranylfarnesyl (GFPP, C_25_) diphosphate. With hexane extraction and GC–MS analysis, only the incubation with FPP yielded a terpene product (Figure 2) that was isolated and identified by one- and two dimensional NMR spectroscopy (Table S2, Supporting Information File 1), EIMS databases and GC retention index as the known sesquiterpene (*Z*)-γ-bisabolene (**5**). Because the two olefinic carbon atoms of its quaternary double bond could not be unambiguously assigned from HMBC data, labelling experiments with (6-^13^C)- and (7-^13^C)FPP [18] were also conducted (Figure S4, Supporting Information File 1). These results characterise the TS from *C. arvum* as a (*Z*)-γ-bisabolene synthase (BbS).

**Scheme 1:**
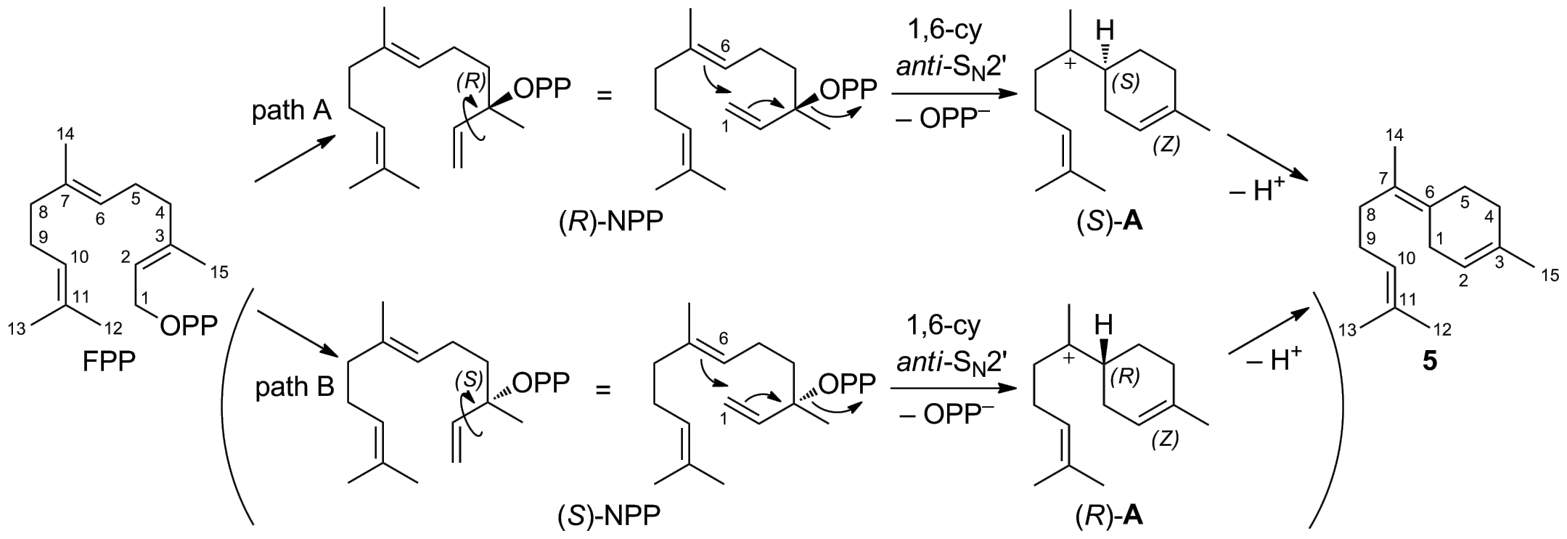
Cyclisation mechanism to **5** involving either the intermediates (*R*)-NPP and (*S*)-**A** (path A) or (*S*)-NPP and (*R*)-**A** (path B). Numbering of carbons in **5** reflect their origin in FPP.

The achiral, monocyclic sesquiterpene **5** is abundant in many essential oils and was reported from different sources such as the liverwort *Dumortiera hirsuta* [19]. Its (*Z*)-configured exocyclic double bond has also attracted the attention of synthetic chemistry for a diastereoselective total synthesis [20–22]. The wide occurrence of **5** is likely connected to the simple biosynthesis from FPP featuring the common bisabolyl cation (**A**) as an intermediate after 1,6-cyclisation (Scheme 1). For this cyclisation, a formal isomerisation of the (*E*)-configured double bond in FPP to the (*Z*)-configured double bond in **A** is needed. To address this problem, a 1,3-suprafacial transposition of OPP to nerolidyl diphosphate (NPP) is usually assumed [23]. This tertiary allylic diphosphate can undergo 1,6-cyclisation after a rotation around the C-2,C-3 single bond. Both NPP and **A** are chiral which raises the question of the active enantiomers in the BbS-catalysed reaction. This problem is challenging since the stereoinformation is destroyed in the final deprotonation step, which prevents any conclusion at the product stage, e.g., by use of enantioselectively labelled substrates [8–10]. If the nucleophilic attack of the C-6,C-7 double bond at the allylic system proceeds with an *anti* stereochemistry (*anti*-S_N_2’ reaction), which is favoured for a concerted process and is also discussed for other cyclisation mechanisms [24–26], the four theoretically possible options for the BbS cyclisation mechanism are narrowed down to two possibilities: Either the reaction takes place via (*R*)-NPP resulting in (*S*)-**A** after ring closure (Scheme 1, path A) or via (*S*)-NPP, which would suggest involvement of (*R*)-**A** (Scheme 1, path B). This stereochemical link between NPP and **A** was also observed in a theoretical docking study with *epi*-isozizaene synthase suggesting (*S*)-NPP and (*R*)-**A** to be included in its cyclisation mechanism [27].

### The absolute configuration of the intermediates nerolidyl diphosphate and the bisabolyl cation

**Figure 3:**
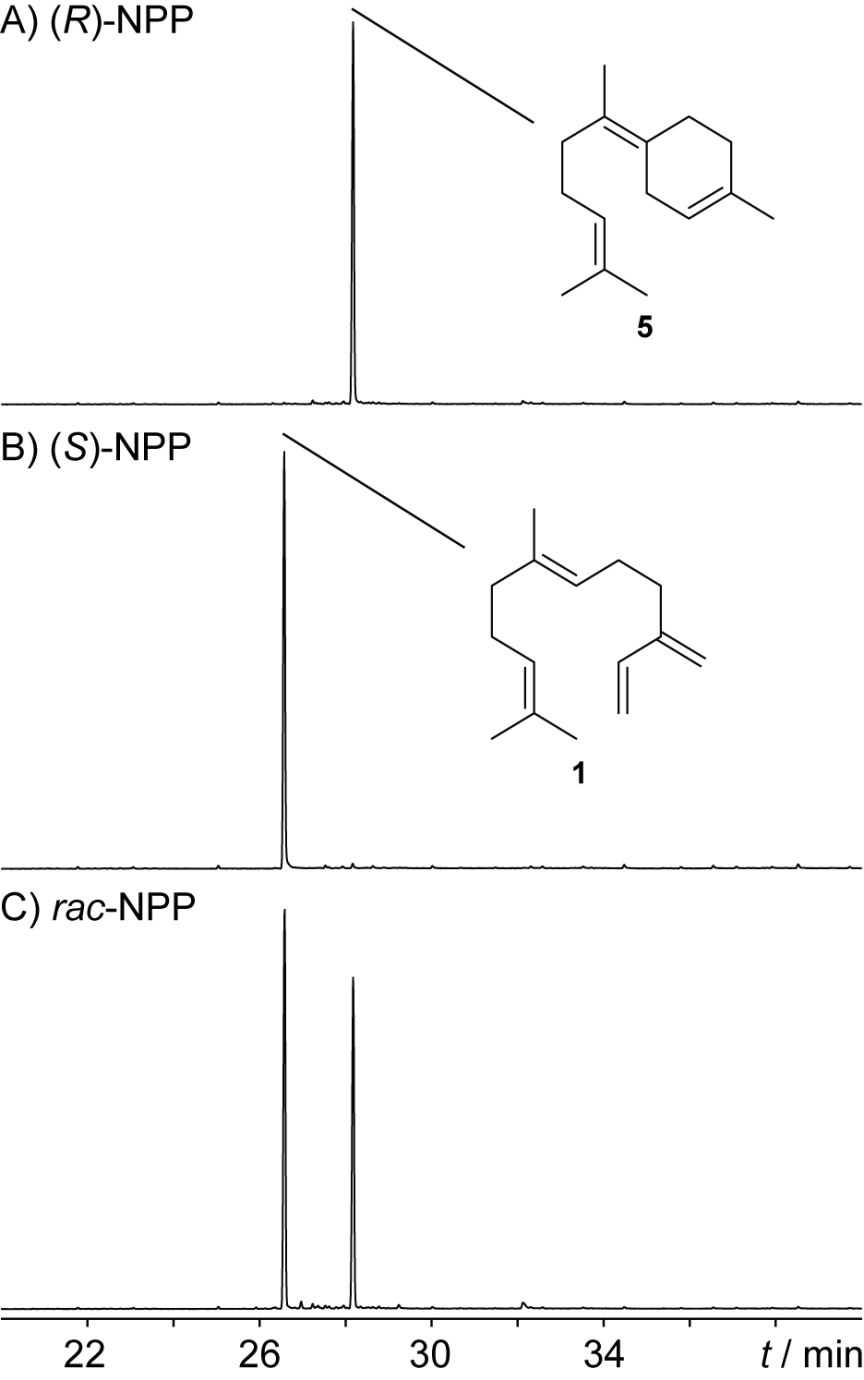
Total ion chromatograms of hexane extracts from incubation experiments with BbS and A) (*R*)-NPP, B) (*S*)-NPP and C) (*rac*)-NPP.

To address this question experimentally, (*R*)- and (*S*)-NPP were synthesised following a known route for enantioselective preparation of nerolidol [28] by Sharpless epoxidation of farnesol in analogy to the reported synthesis of geranyllinaloyl diphosphates [29] (Scheme S1, Supporting Information File 1). Aiming for an easy and unambiguous interpretation of the incubation experiments, the synthesised nerolidol samples (showing moderate to good ee values as judged by Mosher ester analysis of the preceeding epoxides, Figure S5, Supporting Information File 1) were purified by preparative HPLC on a chiral stationary phase to >99% ee for both samples (Figure S6, Supporting Information File 1), before converting them into the NPPs. For comparison, also racemic NPP was synthesised by a Grignard reaction of geranylacetone with vinylmagnesium bromide. The two NPP samples featuring a well-defined stereocentre, and (*rac*)-NPP, were incubated with recombinant BbS, the experiments were extracted with hexane and analysed by GC–MS (Figure 3). A selective product formation was observed for the two enantiomers of NPP, which is surprising in the light of the fact that these reactive tertiary allylic diphosphates were often found to result in a complex mixture of terpene cyclase products and Mg^2+^-catalysed spontaneous hydrolysis products for other TSs [29–30]. While the reaction with (*R*)-NPP leads to BbS’s native product **5**, for (*S*)-NPP formation of the acyclic elimination product (*E*)-β-farnesene (**1**) was observed, which was identified by EI mass spectral library and GC retention index (*I* = 1460 (HP-5MS), lit: *I* = 1459 (HP-5MS) [31]). Incubation with (*rac*)-NPP resulted in a nearly 1:1 mixture of both products, showing that both enantiomers of NPP were converted with similar efficiency.

**Figure 4:**
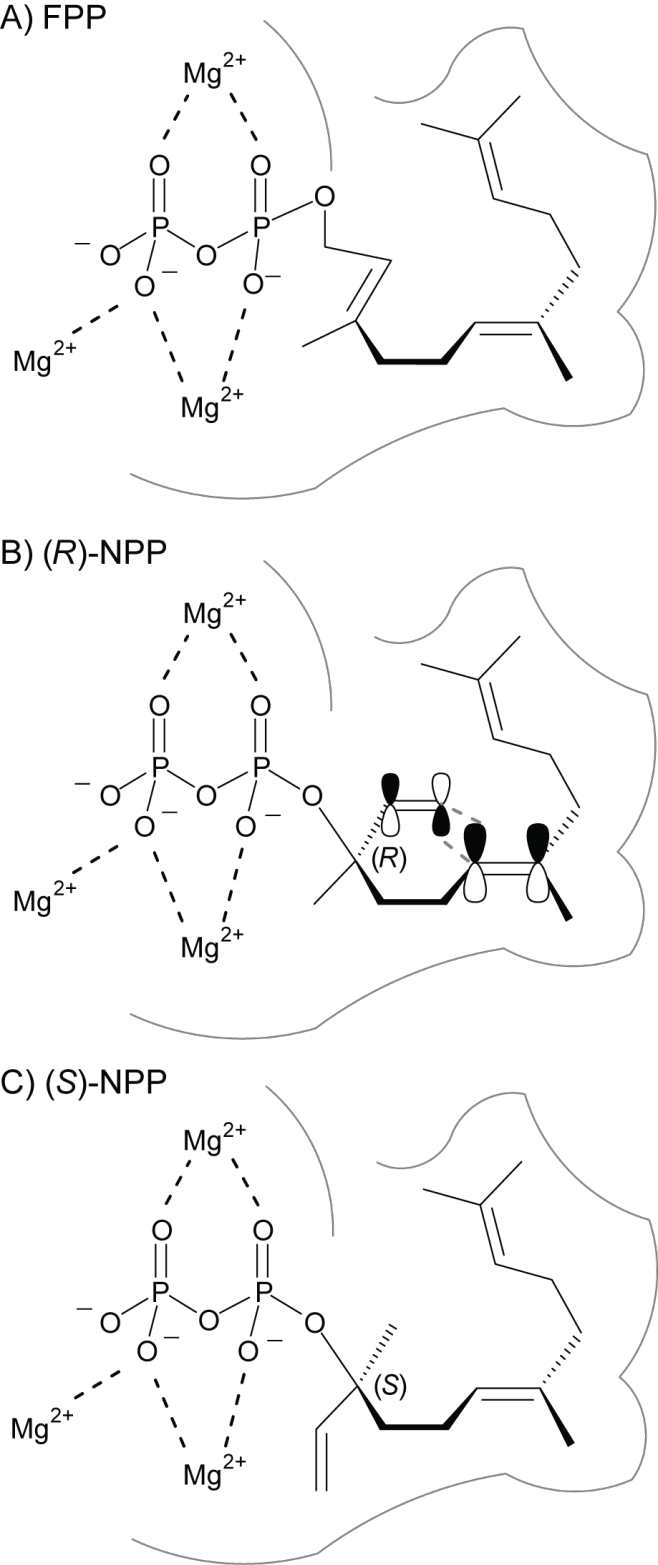
Hypothetical BbS active site comparable conformational folds of A) FPP, B) (*R*)- and C) (*S*)-NPP explaining the selective formation of **5** from (*R*)-NPP by ring closure via (*S*)-**A** and of **1** from (*S*)-NPP by hampering ring closure leading to elimination.

These results clearly rule out (*S*)-NPP, but rationalise (*R*)-NPP as an intermediate in the cyclisation mechanism of BbS, and are in favour of the (*S*)-bisabolyl cation (**A**) to be deprotonated to **5** within the cascade reaction (Scheme 1, path A) [24–26], although the stereochemical link between NPP and **A** could not be demonstrated experimentally in this study. The formation of (*R*)-NPP from FPP as a 1,3-*syn*-allylic rearrangement can be rationalised in a binding mode of FPP, in which OPP is located on a defined face of the C-2,C-3-double bond by the enzyme’s active site (e.g., on top of it, Figure 4A). This migration of OPP to C-3 results in a reorganisation of the resulting structure to a cisoid conformation for the follow up 1,6-ring closure. To explain the astonishing selectivity between the two NPP enantiomers by BbS, different NPP conformations inside the chiral environment of the active site in BbS have to be assumed (Figure 4B + 4C). The architecture of the active site may stay the same in both cases, so a fixed OPP moiety with binding by the trinuclear Mg^2+^ cluster and a comparable folding of the isoprenoid chain in both cases is reasonable. Therefore, the two smallest substituents at the stereocentre formally change their places for the two enantiomers of NPP, representing a minor structural change of the substrate that can be tolerated in the active site. While the binding of (*R*)-NPP leads to a productive conformation that exhibits a close proximity between C-6 and C-1 for ring closure to (*S*)-**A** initiated by OPP abstraction, (*S*)-NPP cannot occupy this conformation for its different stereocentre. Instead, abstraction of the OPP moiety leads to an allylic cation with an unproductive conformation for further ring closure and is thus quenched by abstraction of a proton, presumably by participation of the diphosphate nearby, to give **1**.

## Conclusion

During the course of this work, a new TS from *C. arvum* was characterised as a (*Z*)-γ-bisabolene (**5**) synthase (BbS). Despite its monocyclic achiral structure, the biosynthesis of **5** proceeds via two chiral intermediates, NPP and the bisabolyl cation (**A**), whereas the absolute configuration of the first was addressed experimentally by the synthesis and in vitro incubation of both enantiomers of NPP. These experiments clearly showed the involvement of (*R*)-NPP in the BbS-catalysed reaction, whereas diphosphate was selectively eliminated from (*S*)-NPP by BbS to yield **1**. The selectivity is understandable by the fixed, chiral active site architecture of BbS promoting ring closure only for (*R*)-NPP. In future studies, this experimental approach will not only provide insights into the stereochemical identity of intermediates in cases of achiral terpenes inhibiting any conclusion from the product structure as shown here, but will also deepen our knowledge of general NPP utilisation by sesquiterpene synthases. The chirality of this tertiary diphosphate is currently largely underinvestigated in the characterisation of TSs, even for cascades requiring its involvement.

## Supporting Information

Experimental details of culture conditions, gene cloning, protein purification, incubation experiments, isolation of **5** and HPLC purifications, the amino acid sequence of BbS, a phylogenetic tree with the location of BbS, SDS-PAGE analysis, listed NMR data of **5**, labelling experiments for NMR assignment, synthetic procedures for the NPPs, Mosher ester analysis of epoxides, and chiral GC analysis of nerolidols.

File 1 Experimental part.
