# Supplementary material for: Stereochemical investigations on the biosynthesis of achiral (Z)-γ-bisabolene in Cryptosporangium arvum
Source: Beilstein J Org Chem. 2019 Mar 27;15:789–94. doi: 10.3762/bjoc.15.75 (PMC6444425; doi:10.3762/bjoc.15.75)
Supplement: File 1 — Experimental part. [file Beilstein_J_Org_Chem-15-789-s001.pdf]

BEILSTEIN JOURNAL OF ORGANIC CHEMISTRY

Supporting Information

for

Stereochemical investigations on the biosynthesis of achiral (*Z*)-γ-bisabolene in *Cryptosporangium arvum*

Jan Rinkel and Jeroen S. Dickschat

Beilstein J. Org. Chem. **2019,** *15,* 789–794. doi:10.3762/bjoc.15.75

Experimental part

License and Terms: This is a supporting information file under the terms of the Creative Commons Attribution License (http://creativecommons.org/licenses/by/4.0). Please note that the reuse, redistribution and reproduction in particular requires that the authors and source are credited.
The license is subject to the *Beilstein Journal of Organic Chemistry* terms and conditions: (https://www.beilstein-journals.org/bjoc)

Strains and culture conditions

Cryptosporangium arvum DSM 44712 was obtained from the DSMZ (Braunschweig, Germany) and was grown in Gym 65 medium (10.0 g malt extract, 4.0 g glucose, 4.0 g yeast extract, 1 L water, pH 7.2) at 28 °C. *Escherichia coli* BL21(DE3) was grown in LB medium (10.0 g tryptone, 5.0 g yeast extract, 5.0 g NaCl, 1 L H_2O, pH 7.2) at 37 °C.

Gene cloning

A liquid culture of *C. arvum* was grown for one week and used for the isolation of genomic DNA (gDNA). The cells were harvested by centrifugation (14600 × g), the medium was discarded and the cell pellet was suspended in SET buffer (75 mM NaCl, 25 mM EDTA, 20 mM Tris HCl, pH 8.0, 5 mL). After addition of lysozyme solution (50 mg mL^{-1}, 100 μL), the mixture was incubated at 37 °C for 30 min. Then, proteinase K solution (50 mg mL^{-1}, 100 μL) and 10% SDS (600 μL) were added and the mixture was incubated for 2 h at 55 °C. For extraction, phenol/chloroform/isoamyl alcohol mixture (25:24:1, 5 mL) was used, the phases were mixed and the mixture centrifuged at 14600 × g for 30 min. The upper layer was transferred to a new tube and the DNA was precipitated by the addition of ethanol (0.6 vol). After centrifugation (14600 × g, 1 min), the supernatant was discarded and the DNA was washed with 70% ethanol (1 mL), centrifuged again and dried overnight. The DNA was dissolved in water to a concentration of 1000 ng/μL [1].

The target gene WP_035857999 was amplified by polymerase chain reaction (PCR) using a standard 3-step PCR protocol provided by the supplier of the used Q5 high-fidelity DNA polymerase (New England Biolabs, Ipswich, MA, USA). In this reaction, gDNA of *C. arvum* was used as a template with the primers JR130f and JR130r (Table S1). PCR conditions were: initial denaturation at 98 °C, 30 s; 3-step cycle: 98 °C, 10 s; 70 °C, 30 s; 72 °C, 30 s; repeated 32 times; final elongation at 72 °C, 5 min). The obtained product was elongated with homology arms by a second PCR using the primers JR131f and JR131r under the same PCR conditions mentioned before. Homologous recombination in yeast was done using the elongated PCR products combined with the pYE-Express shuttle vector (linearised by digestion with HindIII and EcoRI) [2] through a standard protocol using LiOAc, polyethylene glycol and salmon sperm DNA [3]. Transformed yeast cultures were grown on SM-URA agar plates (425 mg yeast nitrogen base, 1.25 g ammonium sulphate, 5 g glucose, 192.5 mg nutritional supplement minus uracil, 5 g agar, 250 mL water) at 28 °C for 3 days and colonies were collected to obtain the recombined plasmid using the Zymoprep Yeast Plasmid Miniprep II kit (Zymo Research, Irvine, CA, USA). The isolated plasmid was used for electroporation of *E. coli* BL21(DE3) electrocompetent cells, which were grown overnight at 37 °C on LB agar plates supplied with kanamycin (50 μg mL^{-1}). Single colonies were picked and inoculated in 6 mL LB medium with kanamycin and grown for 8 h at 37 °C to isolate single plasmids. The correct insertion of the target gene was checked by analytical digest with XhoI and PvuII and by sequencing to obtain the plasmid pYE-BbS.

Table S1: Primers used for gene cloning.

Primer	Sequence[a]
JR130f	ATGATCGACGTTCCCGTCCTGC
JR130r	TCATCGGTCCATCCCTTCGAAGATCAG
JR131f	GGCAGCCATATGGCTAGCATGACTGGTGGAATGATCGACGTTCCCGTCCTGC
JR131r	TCTCAGTGGTGGTGGTGGTGGTGCTCGAGTTCATCGGTCCATCCCTTCGAAGATCAG

[a]: Homology arms to pYE-express are underlined.

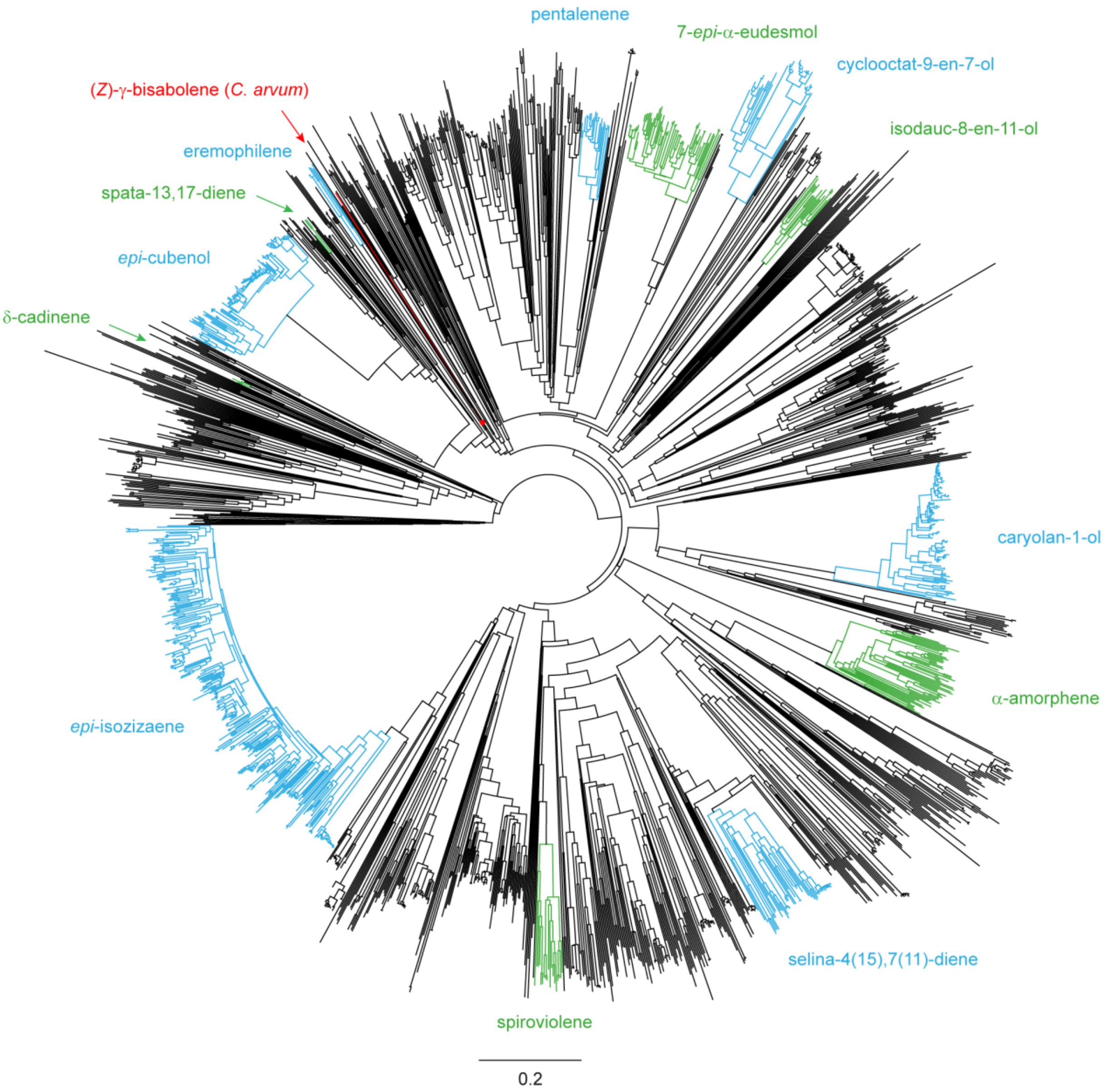

Figure S1: Location of BbS (highlighted in red) in a phylogenetic tree of bacterial terpene synthases (synthases for geosmin and 2-methylisoborneol were omitted). Closest characterised homologs are (% identity): spata-13,17-diene synthase [4] from *Streptomyces xinghaiensis* (30%), δ-cadinene synthase [5] from *S. clavuligerus* (27%), *epi*-cubenol synthase [6] from *S. griseus* (26%) and eremophilene synthase [7] from *Sorangium cellulosum* (25%). A red dot indicates the position of the first junction of BbS towards other terpene synthases.

```
MIDVPVLRVPFPARINPHSTTLGARLRHWLQRSGLATPELNRQFERARFDLLVASLYPAAGAEELRTL
AELVAWMFVYDDHFDVHRLGGSPANAARAADQVSAVLAGAPASGPLLTALSDLRRNRLSAVPAPLRQR
LLGHLDDYCRSLVRELEFRAANRIPAPGAYFDLRMNTFAWPVLADLAEFADGIVLPAAVRDSPEFAAL
LSTSGHLMIVIQDLRSLDRELANGESHNVVLSLREERRCSLPEAVELAHRIFVERLGEFLSRRDAVLV
AFDRLALDDRARRAAASYVVGLEHLLSGHLAWYARTDRYGRAGALESLGGTDDLIFEGMDR
```

Figure S2: Amino acid sequence of BbS (accession number WP_035857999, gene locus tag CRYAR_RS37785). Highly conserved residues and motifs are marked in yellow.

Gene expression and protein purification

The *E. coli* transformant was grown in precultures of LB medium (10 mL) supplied with kanamycin (50 µg/mL final concentration) with shaking overnight at 37 °C. The preculture (1/500) was then used to inoculate main cultures in LB medium with kanamycin at the same concentration and the cells were grown with shaking at 37 °C until OD_{600} = 0.4–0.6 was reached. After cooling the cultures to 18 °C, IPTG (0.4 mM final concentration) was added to the cultures to induce expression. The cultures were shaken at 18 °C overnight and then harvested by centrifugation (8000 × g, 35 min, 4 °C). The medium was removed and the cell pellets were suspended in binding buffer (10 mL/L culture; 20 mM Na_2HPO_4, 500 mM NaCl, 20 mM imidazole, 1 mM $MgCl_2$, pH = 7.4, 4 °C), followed by cell lysis with ultra sonification on ice (50% power, 5 × 1 min). The resulting suspension was centrifuged (14600 × g, 3 × 7 min, 4 °C) and the soluble protein fractions were filtrated and loaded on a Ni^{2+}-NTA affinity chromatography column (Super Ni-NTA Resin, Generon, Slough, UK). The bound protein was washed with binding buffer (2 × 10 mL/L culture) and eluted from the material with elution buffer (2 × 6.25 mL/L culture; 20 mM Na_2HPO_4, 500 mM NaCl, 500 mM imidazole, 1 mM $MgCl_2$, pH = 7.4, 4 °C). Protein containing fractions were analysed by SDS-PAGE (Figure S2) and used for incubation experiments. Approximated typical protein concentrations in the complete elution fraction using this procedure were 2.2 mg/mL, as determined by Bradford assay [8] calibrated to bovine serum albumin.

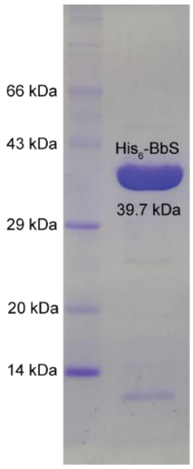

Figure S3: SDS-PAGE analysis of purified recombinant BbS with its calculated molecular weight.

GC–MS analysis

To record GC/MS data, an Agilent (Santa Clara, CA, USA) 7890B GC, which was connected to a 5977A mass detector using a HP5-MS fused silica capillary column (30 m, 0.25 mm i. d., 0.50 μm film) was utilised. GC parameters were 1) inlet pressure: 77.1 kPa, He at 23.3 mL min^{-1}, 2) injection volume: 2 μL or 1 μL, 3) temperature program: 5 min at 50 °C, then increasing at 5 °C min^{-1} or 10 °C min^{-1} to 320 °C, 4) 60 s valve time, and 5) carrier gas: He at 1.2 mL min^{-1}. MS parameters were 1) source: 230 °C, 2) transfer line: 250 °C, 3) quadrupole: 150 °C and 4) electron energy: 70 eV.

Chiral GC analysis

An Agilent GC 7820A GC system equipped with an FID detector and an Agilent Cyclosil-B capillary column (30 m, 0.25 mm inner diameter, 0.25 µm film) was used for chiral GC analysis. For analysis of nerolidol, the GC was programmed as follows: starting from 70 °C, increasing with 2 °C/min to 155 °C, then further increasing with 20 °C/min to 245 °C while holding this temperature for 5 min. Inlet temperature: 250 °C, injection volume: 1 μL, carrier gas: H_2 @ 2.3 mL/min.

NMR spectroscopy

The instruments Bruker (Billerica, MA, USA) Avance I (400 MHz), Avance I (500 MHz), Avance III HD Prodigy (500 MHz) or an Avance III HD Cryo (700 MHz) were used to record NMR data. The obtained spectra were referenced against solvent signals (^{1}H-NMR, residual proton signals: $CDCl_3$ δ = 7.26 ppm, C_6D_6 δ = 7.16 ppm, DMSO-d_6 δ = 2.50 ppm; ^{13}C-NMR: $CDCl_3$ δ = 77.16 ppm, C_6D_6 δ = 128.06 ppm, DMSO-d_6 δ = 39.52 ppm) [9].

Incubation experiments with recombinant BbS and oligoprenyl diphosphates

The diphosphates GPP, FPP, GGPP and GFPP (1 mg each) were dissolved in substrate buffer (1 mL; 25 mM NH_4HCO_3). After dilution with binding buffer (3.5 mL) and incubation buffer (5 mL; 50 mM Tris/HCl, 10 mM $MgCl_2$, 20% glycerol, pH = 8.2), BbS elution fraction (0.5 mL) was added. The reactions were incubated at 28 °C with shaking for 3 h and extracted with hexane (300 μL). The organic layer was dried with $MgSO_4$ and directly analysed by GC–MS. Product formation could only be observed in the experiment with FPP (Figure 2).

Preparative scale isolation of (*Z*)-γ-bisabolene from an FPP incubation with BbS

FPP (60 mg) was dissolved in substrate buffer (20 mL) and the solution was dropped to a slowly stirred mixture of incubation buffer (100 mL), water (50 mL) and BbS elution fraction (50 mL, prepared from 4 L of E. coli expression culture) over 2 h. The reaction mixture was incubated overnight at 28 °C with shaking and extracted with pentane (3 × 50 mL). The combined organic layers were dried with $MgSO_4$ and concentrated under reduced pressure to yield (*Z*)-γ-bisabolene (**5**, 2.0 mg) as a colourless oil after column chromatography on silica gel [pentane].

(*Z*)-γ-bisabolene. TLC [pentane]: R_f = 0.71. GC (HP-5MS): *I* = 1523 [Lit: *I* = 1519 (HP-5MS)] [10]. MS (EI, 70 eV): *m*/*z* (%) = 204 (42), 189 (4), 161 (16), 147 (11), 134 (33), 119 (84), 107 (77), 93 (100), 79 (34), 69 (20), 55 (25), 41 (43), see Figure 2. NMR data are listed in Table S2.

Table S2: NMR data of (*Z*)-γ-bisabolene (**5**) in C_6D_6 recorded at 298 K, cf. literature data [11].

C[a]		^1H[b]	^{13}C[b]
1	CH_2	2.84–2.81 (m, 2H)	29.8
2	CH	5.42–5.39 (m)	121.4
3	C_q	–	134.0
4	CH_2	1.97–1.93 (m, 2H)	31.9
5	CH_2	2.32 (t, $^3J_{H,H}$ = 6.3, 2H)	27.4
6	C_q	–	128.9
7	C_q	–	125.7
8	CH_2	2.14–2.12 (m, 2H)	34.9
9	CH_2	2.14–2.12 (m, 2H)	27.3
10	CH	5.25–5.20 (m)	125.1
11	C_q	–	131.2
12	CH_3	1.67–1.66 (m, 3H)	25.9
13	CH_3	1.56–1.55 (m, 3H)	17.7
14	CH_3	1.71–1.70 (m, 3H)	18.0
15	CH_3	1.64–1.63 (m, 3H)	23.6

[a] Carbon numbering as shown in Scheme 1 of main text. [b] Chemical shifts δ in ppm, multiplicity: s = singlet, t = triplet, m = multiplet, coupling constants *J* are given in Hertz.

Incubation experiments with recombinant BbS and isotopically labelled substrates
The labelled substrates (6-^{13}C)FPP and (7-^{13}C)FPP [12] (1 mg each) were dissolved in substrate buffer (1 mL), diluted with binding buffer (3 mL) and with incubation buffer (5 mL). The reactions were started by adding BbS elution fraction (1 mL) and incubated for 2 h at 28 °C with shaking. The labelled sesquiterpenes were extracted with C_6D_6 (650 µL, 300 µL) and the combined extracts were analysed by GC–MS and NMR.

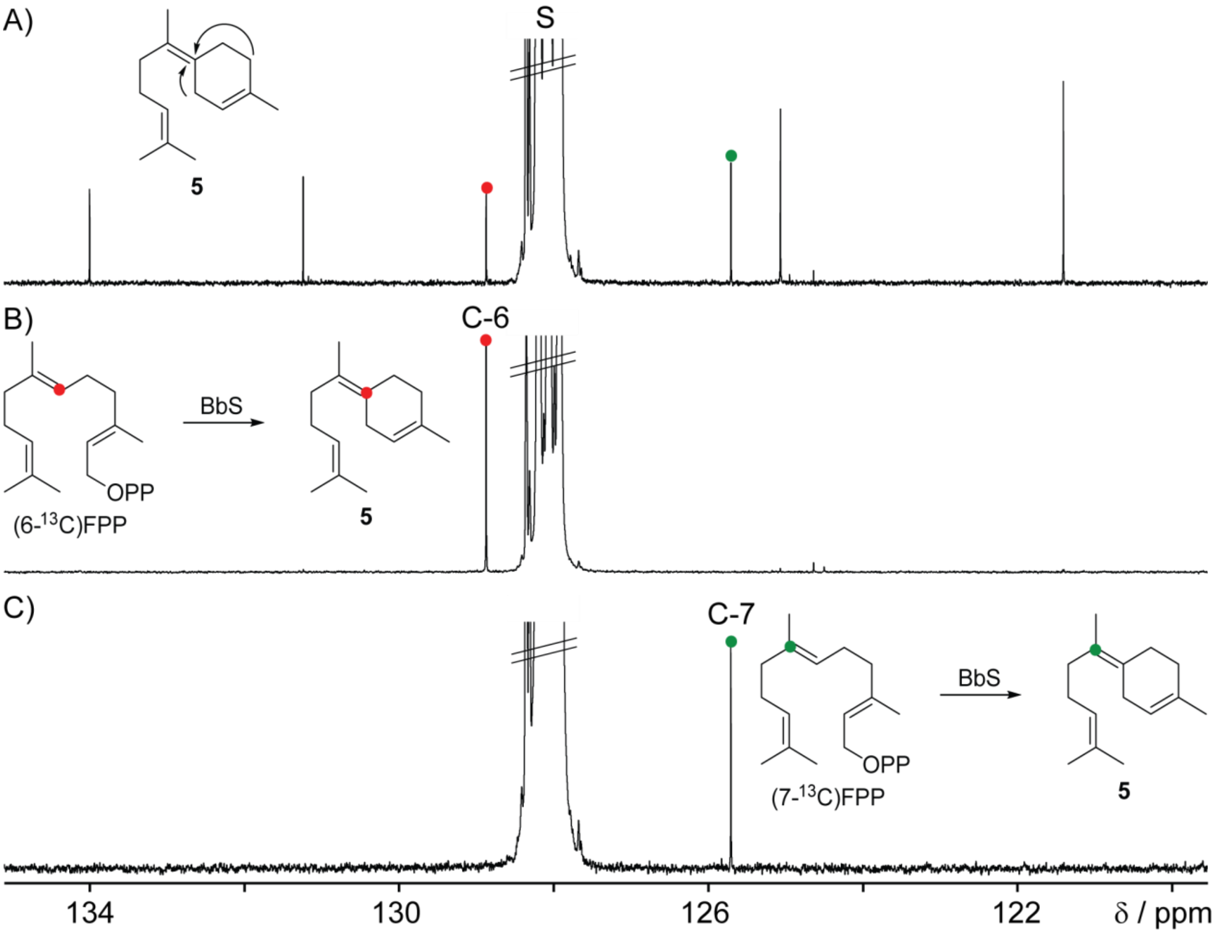

Figure S4: Partial ^{13}C-NMR spectra of A) unlabelled (*Z*)-γ-bisabolene (**5**) with HMBC correlations indicated by arrows leading to questionable assignment of C-6 and C-7, and of labelled **5** arising from incubation experiments of BbS with B) (6-^{13}C)FPP and C) (7-^{13}C)FPP. Coloured dots indicate ^{13}C-labelled carbon atoms. The carbon signal arising from the NMR solvent C_6D_6 is annotated as S.

General synthetic methods

Chemicals were purchased from TCI (Tokyo, Japan), Sigma Aldrich (St. Louis, MO, USA) or Thermo Fisher Scientific (Waltham, MA, USA) and used without further purification. Flash column chromatography was performed with silica Geduran® Si 60 (40 – 63 μm) (Merck, Darmstadt, Germany). Solvents for column chromatography were distilled prior to use. All reactions containing non aqueous solvents or reagents were carried out in flame dried flasks. Argon was used as inert gas. Reaction solvents were dried according to standard procedures. All reactions and chromatographic steps were monitored, if possible, using TLC (silica, Polygram® SIL G/UV254, Macherey-Nagel, Düren, Germany). Staining was done with a solution of phosphomolybdic acid in EtOH (10 g/100 mL).

HPLC conditions

Analytical scale HPLC purifications were carried out using an Azura series HPLC system (Knauer, Berlin, Germany), equipped with a diode array detector DAD 6.1L (190–1020 nm) and a Daicel (Tokyo, Japan) Chiralpak® IA column (5 μm; 4.6 × 250 mm) using an isocratic solvent mixture of [*n*-hexane/ethanol (98:2)] with 1.0 mL min^{-1} (39 bar). The UV–vis absorption was monitored at 210 nm. Observed elution times were: (*S*)-nerolidol: 5.67 min; (*R*)-nerolidol: 7.11 min.

Preparative scale HPLC purifications were performed on an Azura series HPLC system (Knauer) with a multi wavelength detector MWL 2.1L (190–700 nm) using a Daicel Chiralpak® IA column (5 μm, 250 × 20 mm). The solvent mixture [*n*-hexane/ethanol (98:2)] was used at 18 mL min^{-1} (86 bar) and monitoring was done at 210 nm. For the nerolidols, peak recycling was used (2×).

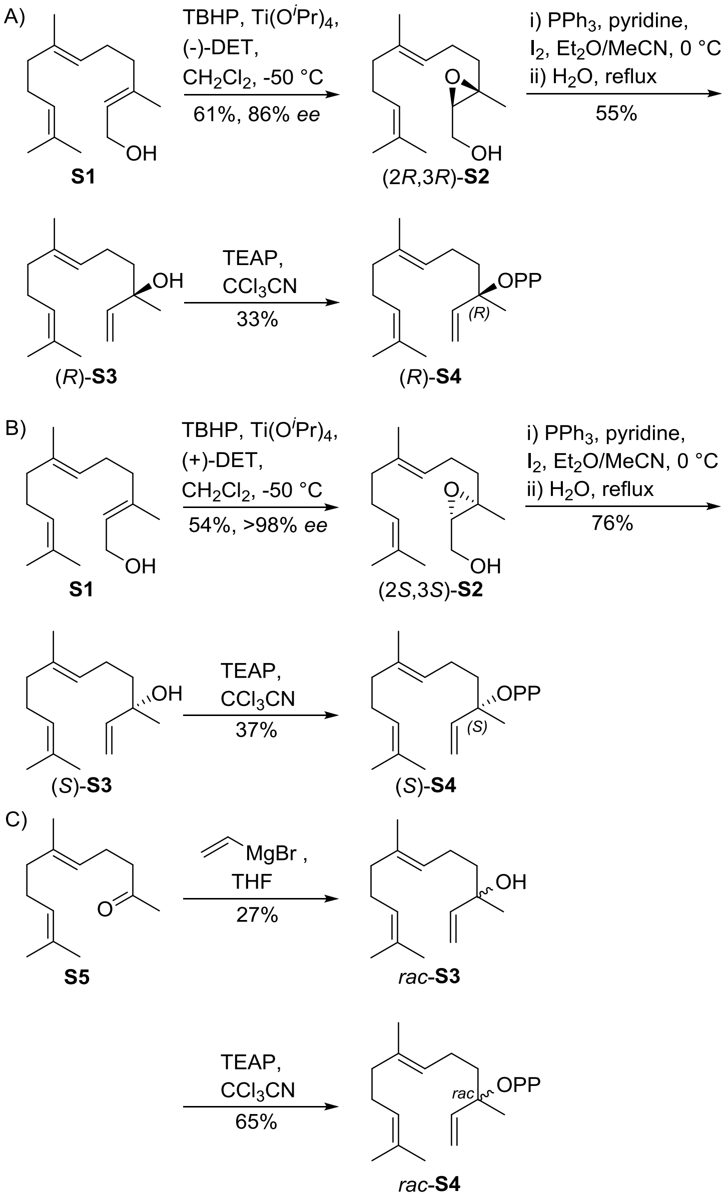

Scheme S1: Synthesis of A) (*R*)-NPP, B) (*S*)-NPP and C) (*rac*)-NPP.

The synthesis of (*R*)- and (*S*)-nerolidyl diphosphate was performed in analogy to the previously utilised route to (*R*)- and (*S*)-geranyllinaloyl diphosphate [13,14].

Synthesis of (2*S*,3*S*)- and (2*R*,3*R*)-3-((*E*)-4,8-dimethylnona-3,7-dien-1-yl)-3-methyloxiran-2-yl)methanol (S2)

In a literature-known procedure [14], (+)-DET (1.13 g, 5.5 mmol, 1.1 equiv) and Ti(O^{i}Pr)$_4$ (1.54 g, 5.4 mmol, 1.08 equiv) were dissolved in CH_2Cl_2 (35 mL) and the mixture was cooled to − 50 °C. A solution of farnesol (**S1**, 1.10 g, 5.0 mmol, 1.0 equiv) in CH_2Cl_2 (10 mL) was added dropwise, before *tert*-butyl hydroperoxide (5.5 M in decane, 2.0 mL, 11.0 mmol, 2.2 equiv) was added. The reaction mixture was stirred for 2 h at −50 °C and hydrolysed by addition of 10% tartaric acid solution (10 mL). The reaction mixture was stirred for 2 h without cooling. The organic phase was washed with H_2O (50 mL) and the aqueous phase was extracted with CH_2Cl_2 (3 × 20 mL). The combined organic layers were dried with $MgSO_4$ and the solvent was removed under reduced pressure. The crude product was dissolved in Et_2O

(30 mL) and cooled to 0°C, followed by addition of 1 M NaOH solution (15 mL). The reaction mixture was stirred for 40 min, the phases were separated and the aqueous phase was extracted with Et_2O (3 × 10 mL). The combined organic phases were washed with saturated NH_4Cl solution and brine followed by drying with $MgSO_4$. The solvent was removed under reduced pressure. Column chromatography on silica gel [cyclohexane:EtOAc (8:1->5:1)] resulted in the (2*S*,3*S*) epoxy alcohol **S2** (0.65 g, 2.7 mmol, 54%, >98% ee) as colourless oil. The same procedure was used to convert farnesol (10.0 mmol) to (2*R*,3*R*)-**S2** (1.45 g, 6.1 mmol, 61%, 86% ee) using (–)-DET. Mosher ester analyses [15] for the determination of the enantiomeric excesses were performed by dissolving 0.2 µL of each epoxy alcohol in $CDCl_3$ (100 µL). Then, pyridine (1 µL) and (*S*)-MTPA-Cl (1 µL) were added. The reaction mixture was stirred for 30 min, diluted with $CDCl_3$ (400 µL) and analysed by ^{1}H-NMR (Figure S5).

(2*S*,3*S*)-S2: ^{1}H-NMR (500 MHz, $CDCl_3$): δ = 5.12-5.05 (m, 2H, 2xCH), 3.86-3.79 (m, 1H, 0.5xCH_2), 3.71-3.64 (m, 1H, 0.5xCH_2), 2.97 (dd, $^3J_{H,H}$ = 6.7 Hz, $^3J_{H,H}$ = 4.3 Hz, 1H, CH), 2.13-2.02 (m, 4H, 2xCH_2), 2.01-1.95 (m, 2H, 1xCH_2), 1.88-1.83 (m, 1H, OH), 1.73-1.65 (m, 1H, 0.5xCH_2), 1.67 (s, 3H, CH_3), 1.60 (s, 3H, CH_3), 1.59 (s, 3H, CH_3), 1.51-1.44 (m, 1H, 0.5xCH_2), 1.30 (s, 3H, CH_3) ppm. ^{13}C-NMR (126 MHz, $CDCl_3$): δ = 135.9 (C_q), 131.6 (C_q), 124.3 (CH), 123.3 (CH), 63.1 (CH), 61.6 (CH_2), 61.3 (C_q), 39.8 (CH_2), 38.6 (CH_2), 26.8 (CH_2), 25.8 (CH_3), 23.7 (CH_2), 17.8 (CH_3), 16.9 (CH_3), 16.1 (CH_3) ppm. TLC [cyclohexane/EtOAc (2:1)]: R_f = 0.35. GC (HP-5MS): *I* = 1813. MS (EI, 70 eV): *m*/*z* (%) = 189 (3), 177 (9), 159 (2), 150 (5), 135 (7), 121 (18), 109 (40), 107 (20), 95 (19), 93 (22), 81 (72), 69 (100), 55 (19), 43 (35), 41 (55). $[\alpha]_D^{20}$ = –5.2° (*c* = 1.0, $CHCl_3$). Lit: $[\alpha]_D^{24}$ = –5.7° (*c* = 1.08, $CHCl_3$) [16].

(2*R*,3*R*)-S2: $[\alpha]_D^{20}$ = +5.8° (*c* = 1.0, $CHCl_3$). Lit: $[\alpha]_D^{20}$ = +5.8° (*c* = 1.05, $CHCl_3$) [17]. Spectroscopic data as for the (2*S*,3*S*) enantiomer.

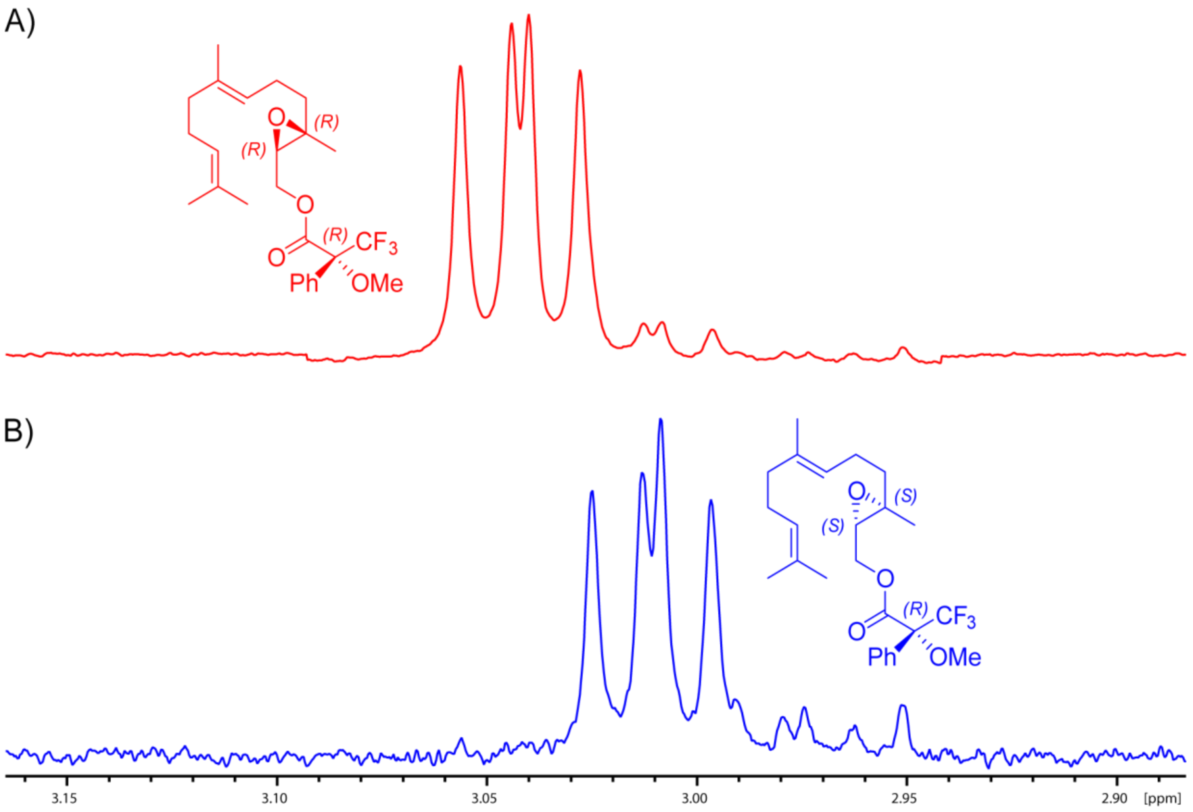

Figure S5: Mosher ester analysis of **S2**. Partial ^{1}H-NMR spectra of the reaction product of (*S*)-MTPA-Cl with A) (2*R*,3*R*)-**S2** and B) (2*S*,3*S*)-**S2** showing the signal for H-2. Integration gave an approximated enantiomeric excess of 86% for (2*R*,3*R*)-**S2** and >98% for (2*S*,3*S*)-**S2**.

Synthesis of (*S*)- and (*R*)-nerolidol (S3)

Following a known procedure [14], (2*S*,3*S*)-**S2** (629 mg, 2.64 mmol, 1.0 equiv) was dissolved in Et_2O:CH_3CN (5:3, 21 mL). The solution was cooled to 0 °C and PPh_3 (2.08 g, 7.92 mmol, 3.0 equiv), pyridine (835 mg, 10.55 mmol, 4.0 equiv) and I_2 (1.01 g, 3.96 mmol, 1.5 equiv) were added sequentially. Stirring was continued for 2 h at 0 °C and then H_2O (47.5 μL, 2.64 mmol, 1.0 equiv) was added. The reaction mixture was heated to reflux for 12 h with stirring. The reaction mixture was quenched by addition of 20% $Na_2S_2O_3$ solution (5.3 mL) and saturated aqueous $NaHCO_3$ solution (5.3 mL). The aqueous phase was extracted with Et_2O (3 × 20 mL), and the combined organic layers were washed with 0.5 M HCl (10 mL) and saturated aqueous $NaHCO_3$ solution (10 mL), dried with $MgSO_4$ and concentrated under reduced pressure. Column chromatography on silica gel [cyclohexane/EtOAc (20:1)] resulted in (*S*)-nerolidol (**S3**, 447 mg, 2.01 mmol, 76%) as a colourless oil. The same procedure was used to convert (2*R*,3*R*)-**S2** (1.43 g, 5.99 mmol) into (*R*)-nerolidol (**S3**, 736 mg, 3.32 mmol, 55%).

(*S*)-Nerolidol. ^{1}H-NMR (700 MHz, $CDCl_3$): δ = 5.91 (dd, $^3J_{H,H}$ = 17.3 Hz, $^3J_{H,H}$ = 10.7 Hz, 1H, CH), 5.21 (dd, $^3J_{H,H}$ = 17.3 Hz, $^3J_{H,H}$ = 1.3 Hz, 1H, 0.5xCH_2), 5.15-5.12 (m, 1H, CH), 5.10-5.06 (m, 1H, CH), 5.06 (dd, $^3J_{H,H}$ = 10.8 Hz, $^3J_{H,H}$ = 1.3 Hz, 1H, 0.5xCH_2), 2.09-1.95 (m, 6H, 3xCH_2), 1.68 (s, 3H, CH_3), 1.62-1.53 (m, 2H, CH_2), 1.60 (br s, 6H, 2xCH_3), 1.28 (s, 3H, CH_3) ppm. ^{13}C-NMR (176 MHz, $CDCl_3$): δ = 145.2 (CH), 135.7 (C_q), 131.6 (C_q), 124.4 (CH), 124.4 (CH), 111.8 (CH_2), 73.6 (C_q), 42.2 (CH_2), 39.8 (CH_2), 28.0 (CH_3), 26.8 (CH_2), 25.8 (CH_3), 22.9 (CH_2), 17.8 (CH_3), 16.2 (CH_3) ppm. TLC [cyclohexane/EtOAc (20:1)]: R_f = 0.21. GC (HP-5MS): *I* = 1564. MS (EI, 70 eV): *m*/*z* (%) = 204 (3), 189 (6), 161 (23), 148 (4), 136 (28), 123 (18), 121 (20), 119 (14), 107 (42), 93 (70), 81 (40), 69 (100), 55 (30), 43 (39), 41 (55). $[\alpha]_D^{23}$ = +4.9° (*c* = 1.0, $CHCl_3$). Lit: $[\alpha]_D^{22}$ = +9.6° [18].

(*R*)-Nerolidol. $[\alpha]_D^{22}$ = –10.4° (*c* = 0.57, $CHCl_3$). Lit: $[\alpha]_D^{23}$ = –12.5° (*c* = 0.02, $CHCl_3$) [19]. Spectroscopic data as for the (*S*) enantiomer.

Both samples of nerolidol were additionally purified by chiral HPLC to increase the enantiomeric excess. The resulting samples were analysed for enantiomeric purity on chiral GC phase and both exhibited >99% ee (Figure S6).

Synthesis of (*rac*)-nerolidol

Geranylacetone (**S5**, 389 mg, 2.0 mmol, 1.0 equiv) was dissolved in THF (9 mL) and the solution was cooled to 0 °C. Then, a solution of vinylmagnesium bromide (2.3 mL; 1 M in THF, 2.3 mmol, 1.15 equiv) was added dropwise. The reaction mixture was stirred for 3 d at room temperature. With TLC control showing poor conversion, two more additions of vinylmagnesium bromide solution (2 × 0.25 equiv) were conducted and the mixture was heated to 50 °C. The reaction mixture was quenched by water (10 mL) and the aqueous phase was extracted with Et_2O (2 × 10 mL). The combined organic layers were dried with $MgSO_4$ and concentrated under reduced pressure to yield (*rac*)-nerolidol (**S3**, 120 mg, 0.54 mmol, 27%) as a colourless oil after column chromatography on silica gel [cyclohexane/EtOAc (20:1)].

(*rac*)-Nerolidol. Spectroscopic data as for the (*S*) enantiomer.

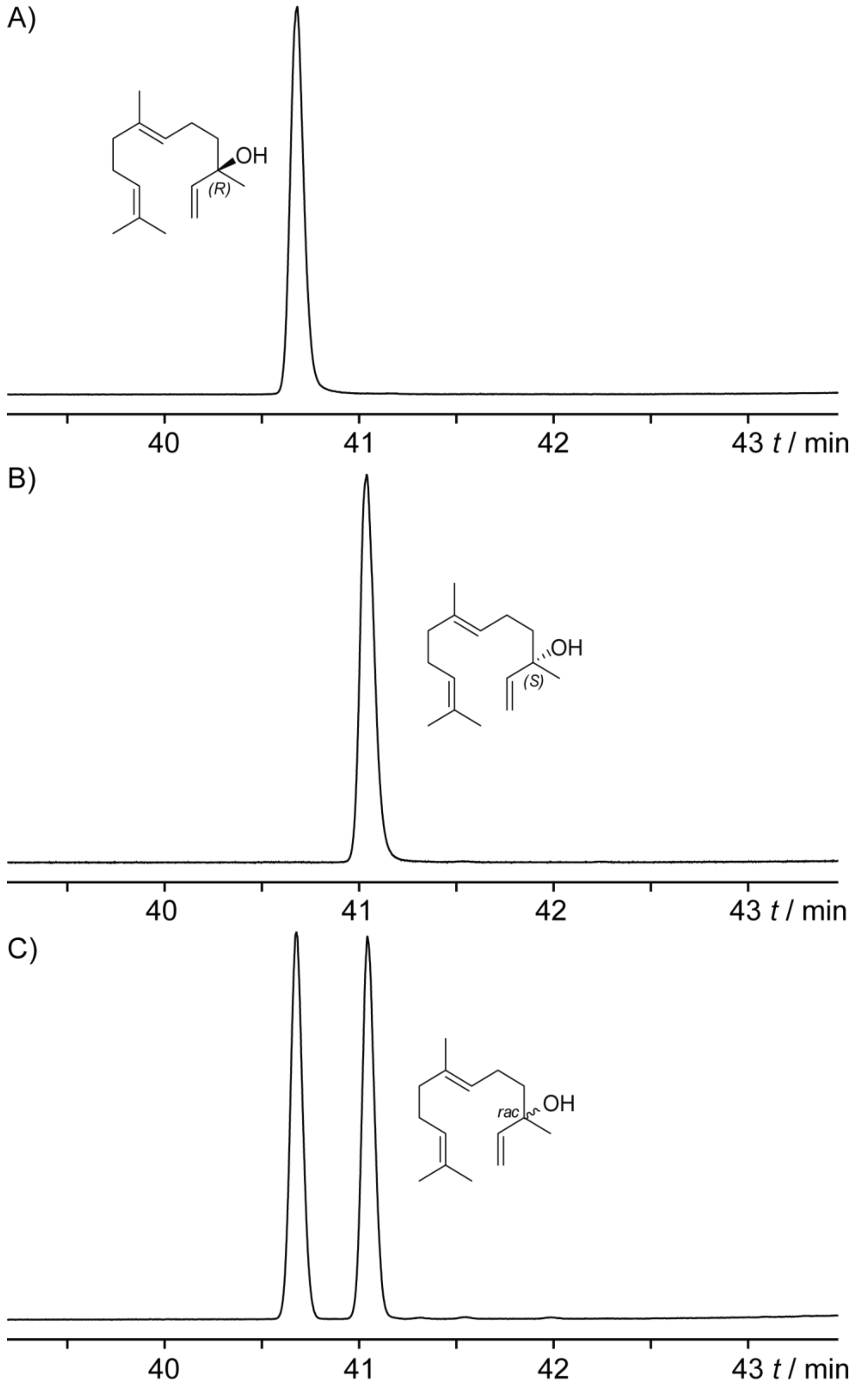

Figure S6: Chiral GC analysis of NPP precursors A) (*R*)-nerolidol and B) (*S*)-nerolidol after chiral HPLC purification together with C) (*rac*)-nerolidol.

Synthesis of (*S*)-, (*R*)-, and (*rac*)-nerolidyl diphosphate (NPP, S4)

In a direct phosphorylation procedure [20,21], bis-triethylammonium phosphate (TEAP) solution (0.76 mL; prepared by adding 1.82 mL of solution A (2.5 mL phosphoric acid, 9.4 mL MeCN) to 3 mL of solution B (11 mL triethylamine, 10 mL MeCN)) was added to a solution of (*S*)-nerolidol (**S3**, 67 mg, 0.30 mmol) in CCl_3CN (0.76 mL) at room temperature. After 2 more

additions of TEAP solution (2 × 0.76 mL) in a 5 min interval, the reaction mixture was chromatographed on silica gel [^{i}PrOH/25% NH_3/H_2O (6:2.5:0.5)] to yield (*S*)-NPP (**S4**, 43 mg, 0.11 mmol, 37%) as a white solid after lyophilisation. Under the same conditions, (*R*)-nerolidol (61 mg, 0.28 mmol) was converted to (*R*)-NPP (**S4**, 35 mg, 0.09 mmol, 33%) and (*rac*)-nerolidol (55 mg, 0.24 mmol) to (*rac*)-NPP (**S4**, 60 mg, 0.16 mmol, 65%).

(*S*)-NPP. ^{1}H-NMR (500 MHz, DMSO-d$_6$): δ = 6.00 (dd, $^3J_{H,H}$ = 17.4 Hz, $^3J_{H,H}$ = 10.9 Hz, 1H, CH), 5.12-5.03 (m, 3H, 2xCH + 0.5xCH_2), 4.97 (d, $^3J_{H,H}$ = 10.9 Hz, 1H, 0.5xCH_2), 2.05-1.86 (m, 6H, 3xCH_2), 1.80-1.72 (m, 1H, 0.5xCH_2), 1.63 (s, 3H, CH_3), 1.59-1.52 (m, 1H, 0.5xCH_2), 1.55 (s, 3H, CH_3), 1.54 (s, 3H, CH_3), 1.43 (s, 3H, CH_3) ppm. ^{13}C-NMR (126 MHz, DMSO-d$_6$): δ = 134.1 (C_q), 130.7 (C_q), 124.5 (CH), 124.2 (CH), 124.2 (CH), 111.9 (CH_2), 67.0 (C_q), 40.9 (CH_2), 40.9 (CH_2), 26.2 (CH_2), 25.5 (CH_3), 24.0 (CH_3), 22.3 (CH_2), 17.6 (CH_3), 15.8 (CH_3) ppm. ^{31}P-NMR (202 MHz, DMSO-d$_6$): δ = -9.8 (d, $^2J_{P,P}$ = 14.5 Hz, 1P), -13.2 (d, $^2J_{P,P}$ = 14.5 Hz, 1P) ppm.

(*R*)-NPP. Spectroscopic data as for the (*S*) enantiomer.

(*rac*)-NPP. Spectroscopic data as for the (*S*) enantiomer.

Incubation experiments using (*R*)-, (*S*)-, and (*rac*)-NPP and BbS

The NPPs (0.05 mg each) were dissolved in substrate buffer (100 µL) and added to a mixture of BbS elution fraction (0.4 mL) with incubation buffer (0.5 mL). The reaction mixtures were incubated for 30 min at 28 °C and extracted with hexane (200 µL). The organic phase was dried with $MgSO_4$ and analysed by GC–MS.

References

1. Neumann, B.; Pospiech, A.; Schairer, H. U. *Trends Genet.,* **1992,** *8,* 332–333.
2. Dickschat, J. S.; Pahirulzaman, K. A. K.; Rabe P.; Klapschinski, T. A. *ChemBioChem,* **2014,** *15,* 810–814.
3. Giets, R. D.; Schiestl, R. H. *Nat. Protoc.,* **2007,** *2,* 31–34.
4. Rinkel, J.; Lauterbach, L.; Dickschat, J. S. *Angew. Chem. Int. Ed.,* **2017,** *56,* 16385–16389.
5. Hu, Y.; Chou, W. K. W.; Hopson, R.; Cane, D. E. *Chem. Biol.,* **2011,** *18,* 32–37.
6. Cane, D. E.; Tandon, M.; Prabhakaran, P. C. *J. Am. Chem. Soc.,* **1993,** *115,* 8103–8106.
7. Schifrin, A.; Ly, T. T. B.; Günnewich, N.; Zapp, J.; Thiel, V.; Schulz, S.; Hannemann, F.; Khatri, Y.; Bernhardt, R. *ChemBioChem,* **2015,** *16,* 337–344.
8. Bradford, M. M. *Anal. Biochem.,* **1976,** *72,* 248–254.
9. Fulmer, G. R.; Miller, A. J. M.; Sherden, N. H.; Gottlieb, H. E.; Nudelman, A.; Stoltz, B. M.; Bercaw, J. E.; Goldberg, K. I. *Organometallics,* **2010,** *29,* 2176–2179.
10. Asuming, W. A.; Beauchamp, P. S.; Descalzo, J. T.; Dev, B. C.; Dev, V.; Frost, S.; Ma, C. W. *Biochem. Syst. Ecol.,* **2005,** *33,* 17–26.
11. Saritas, Y.; Bülow, N.; Fricke, C.; König, W. A.; Muhle, H. *Phytochemistry,* **1998,** *48,* 1019–1023.
12. Rabe, P.; Barra, L.; Rinkel, J.; Riclea, R.; Citron, C. A.; Klapschinski, T. A.; Janusko, A.; Dickschat, J. S. *Angew. Chem. Int. Ed.,* **2015,** *54,* 13448–13451.
13. Rinkel, J.; Rabe, P.; Chen, X.; Köllner, T. G.; Chen, F.; Dickschat, J. S. *Chem. Eur. J.,* **2017,** *23,* 10501–10505.
14. Le, T. C.; Chauhan, K. R. *Nat. Prod. Commun.,* **2014,** *9,* 297–298.
15. Hoye, T. R.; Jeffrey, C. S.; Shao, F. *Nat. Protoc.,* **2007,** *2,* 2451–2458.
16. Egami, H.; Oguma, T.; Katsuki, T. *J. Am. Chem. Soc.,* **2010,** *132,* 5886–5895.
17. Marshall, J. A.; Hann, R. K. *J. Org. Chem.,* **2008,** *73,* 6753–6757.
18. Ohloff, G.; Schulte-Elte, K.-H.; Willhalm, B. *Helv. Chim. Acta* **1964,** *47,* 602–626.
19. Cane, D. E.; Ha, H.-J.; McIlwaine, D. B.; Pascoe, K. O. *Tetrahedron Lett.,* **1990,** *31,* 7554–7554.
20. Thulasiram, H. V.; Phan, R. M.; Rivera, S. B.; Poulter, C. D. *J. Org. Chem.,* **2006,** *71,* 1739–1741.
21. Keller, R. K.; Thompson, R. *J. Chromatogr. A,* **1993,** *645,* 161–167.
